# Prognostic and therapeutic potential of Adenylate kinase 2 in lung adenocarcinoma

**DOI:** 10.1038/s41598-019-53594-4

**Published:** 2019-11-28

**Authors:** Huibin Liu, Yan Pu, Quhai Amina, Qiang Wang, Mengmeng Zhang, Jianzhong Song, Jun Guo, Mahmut Mardan

**Affiliations:** 1 Institute of cancer control, Affiliated Tumor Hospital of Xinjiang Medical University, Urumqi, China; 2 Department of pulmonary oncology, Affiliated Tumor Hospital of Xinjiang Medical University, Urumqi, China; 3 School of pharmacy, Xinjiang Medical University, Urumqi, China

**Keywords:** Non-small-cell lung cancer, Non-small-cell lung cancer, Mitophagy, Mitophagy

## Abstract

Adenylate kinase 2 (AK2), an isoenzyme of the AK family, may have momentous extra-mitochondrial functions, especially in tumourigenesis in addition to the well-known control of energy metabolism. In this study, we provided the first evidence that AK2 is overexpressed in lung adenocarcinoma. The positive expression of AK2 is associated with tumor progression, and poor survival in patients with pulmonary adenocarcinoma. Knockdown of AK2 could suppress proliferation, migration, and invasion as well as induce apoptosis and autophagy in human lung adenocarcinoma cells. Remarkably, silencing AK2 exerted the greater tumor suppression roles when combined with hydroxychloroquine, an effective autophagy inhibitor, *in vitro* and in xenografts mouse models. Our data have probably provided preclinical proof that systematic inhibition of AK2 and autophagy could be therapeutically effective on lung cancer.

## Introduction

Lung cancer is one of the most common malignancies and ranks the first leading cause of cancer death worldwide^1^. According to “2015 Cancer Statistics in China”, lung cancer is also the biggest cause of death from cancer in China^2^. Non-small-cell lung cancer (NSCLC) accounts for 85% of all lung cancer cases and approximately half of NSCLC are lung adenocarcinoma (LAD)^3^. In the recent ten years, great progress has been made in the treatment of LAD with molecular target therapy such as drugs targeting the epidermal growth factor receptor (EGFR) mutation or the anaplastic lymphoma kinase (ALK) fusion^4,5^. However, the prognosis of LAD is still unsatisfactory due to drug resistance, recurrence and metastasis^6^. Therefore, discovery of novel, functional gene is paramount, and a better understanding of molecular mechanisms underlying tumor progression may facilitate the development of effective therapeutic strategies for LAD.

Adenylate kinase (AK) is an ubiquitous enzyme that catalyses the nucleotide phosphoryl exchange reaction^7^. To date, eight isoenzymes of the AK family comprising AK1-AK8 have been identified in vertebrates forming a circuit in monitoring cell energy metabolism and AMP signal transduction^8^. Among them, AK2 is a special enzyme expressed in the mitochondrial intermembrane space, and the unique subcellular localization suggests that AK2 plays an unique role in energy metabolism and transfer by regulating the ATP/ADP rate between the cytoplasma matrix and the mitochondria^9^. The single isoform of adenylate kinase present in bacteria and lower eukaryotes is essential for life^10–12^. In a previous study, Drosophila adenylate kinase isozyme 2 knockout was reported to cause developmental lethality at the larval stage in Drosophila melanogaster^13^. In addition, deregulation of AK2 function could be involved in the alteration of mitochondrial metabolism and, consequently, in the development of human disease^14^. Two other studies showed that AK2 is the gene responsible for reticular dysgenesis, a human disease that is characterized by severe combined immunodeficiency and deafness^15,16^. Therefore, mitochondrial AK2 may play an important role in hematopoietic differentiation and ontogenesis. Moreover, its dysregulation was also observed in human cancers and amyotrophic lateral sclerosis diseases^17–20^. Thus, besides well-known roles in energy metabolism, AK2 may have momentous extra-mitochondrial functions, especially in tumourigenesis. Hyunjoo *et al*. showed that AK2 deficiency could enhance the proliferation of breast cancer MCF-7 and C33A cells *in vitro* and induce tumor formation in a xenograft assay^21^. In a report published in 1980, AK was proposed to exhibit lower activities in normal fetal lungs than in adult lungs, being deficient in pulmonary cancers, and was regarded as the only reliable “negative” indicator of pulmonary neoplasia thus far identified^22^. Although the earlier works demonstrated a biochemical difference of AK2 in lung cancer cases, the molecular events involved in regulating tumor growth and metastasis remained unknown.

In this study, we determined the contribution of AK2 to LAD progression. We found that AK2 expression had meaningful difference in LAD tissues, and was critical for the ability of migration and invasion of LAD cells *in vitro*. Moreover, it was found that autophagy inhibitor may potentially coordinate with AK2 knockdown in treatment of LAD.

## Materials and Methods

### Chemicals and antibodies

Dulbecco’s modified Eagle’s medium (DMEM), fetal bovine serum (FBS), trypsin, penicillin and streptomycin were obtained from GIBCO (Carlsbad, CA). Trizol (Invitrogen, Carlsbad, CA) and propidium iodide (PI) (Sigma-Aldrich, St Louis, MO) were purchased. MatrigelTM matrix (BD Biosciences, San Jose, CA), transwell Boyden chamber system and 6-well Ultra-low Adherence plates (Corning life Sciences, Wilkes Barre, PA) were also used. A tandem monomeric RFP-GFP-tagged LC3 (tfLC3) was purchased from Shanghai Genechem Company Ltd., China. Hoechest-33342 stain was purchased from Beijing Suolaibao Biotechnology Co., Ltd. (Beijing, China). Antibodies specific to AK2 and GAPDH were purchased from Santa Cruz Biotechnology (SantaCruz, CA, USA; No. sc-374095 and sc-32233). Antibodies specific to E-cadherin, Vimentin and LC3I/II were obtained from Cell Signaling Technology (CST, MA, USA; No. 14472, 5741 and 12741). Antibodies specific to Twist and p62 were supplied by Abcam (Cambridge, UK; No. ab50581 and ab56416), Antibody against Snail2 was provided by PLLABS (BC, Canada; No. PL0305187). Hydroxychloroquine (HCQ) was obtained from J&K chemical Ltd., China. All other chemicals were classified as analytical grade reagents.

### Tissue samples and clinical data collection

Material was harvested from 345 frozen carcinoma tissues and 80 para-carcinoma tissues isolated from lung adenocarcinoma patients who were hospitalized in the Affiliated Tumor Hospital of Xinjiang Medical University (Urumqi, Xinjiang, China) between January 2012 and August 2016. None of the patients had received preoperative radiotherapy or chemotherapy. All samples were pathologically confirmed as LAD by two pathologists. Informed consent was obtained from all patients and this study was approved by the Review Board of Hospital Ethics Committee of Affiliated Tumor Hospital of Xinjiang Medical University. All experiments were performed in accordance with relevant guidelines and regulations. Clinicopathological characteristics of LAD patients are shown in Table 1.

**Table 1:** The relationship between AK2 expression and the clinicopathological features of 345 LAD patients.

Characteristics	AK2 High (n = 273)	AK2 low (n = 72)	P value
**Age**, **year**			
≤60	136	38	P = 0.655
>60	137	34	
**Gender**			
Male	121	37	P = 0.284
Female	152	35	
**Tumor diameter**			
<3	140	44	P = 0.137
≥3	133	28	
**AJCC stage**			
I/II	152	59	P < 0.001
III/IV	121	13	
**Smoking**			
Yes	84	28	P = 0.191
No	189	44	
**Lymphatic metastasis**			
Yes	145	21	P < 0.001
No	128	51	
**pleural metastasis**			
Yes	111	23	P = 0.177
No	162	49	
**Subtype**			
Acinar	226	54	P = 0.007
Solid	55	26	
Lepidic	20	3	
Papillary	33	16	
Mucinous	16	3	

### Cell lines and cell culture

Cell culture were as described previously^23^. Four human lung adenocarcinoma cell lines (H1299, A549, 95D and H460) and human normal bronchial epithelial cell line (16HBE) were obtained from the American Type Culture Collection (ATCC). All cells were cultured in Dulbecco’s modified Eagle medium (DMEM) supplemented with 10% fetal bovine serum (FBS), 100 U/mL penicillin, and 100 mg/mL streptomycin at 37 °C in a humidified atmosphere with 5% CO_2_.

### RT-PCR

Total RNA was reversely transcribed using a PrimeScript RT Mix (Takara, China) in accordance with the manufacturer’s instructions. Following RT-PCR amplification, melting curve analysis was executed to identify the specific generation of the PCR product with glyceraldehyde 3-phosphate dehydrogenase (GAPDH) used as internal controls. Fold changes for aberrant expression were calculated using the 2–ΔΔCt method. Reactions were performed in 20 μL system including SYBR Select Master Mix(Thermo Fisher Scientific) and 40 ng cDNA with the following reaction conditions: 95 °C 10 minute, 40 cyclesof 95 °C 30 sec, 58 °C 1 minute, 72 °C 1 minute. The primer used for the amplification of the human AK2 gene was as follows: forward primer, 5′-GCAGAACCCGAGTATCCTAAAGG, and reverse primer, 5′-TTCCCAGCATCCATAGTTGCC. GAPDHwas used as the housekeeping gene with forward primer 5′-TTCGACAGTCAGCCGCATCTTCTT and reverseprimer 5′-ACCAAATCCGTTGACTCCGACCTT. Delta-delta Ct values were used to determine their relative expression as fold changes, as previously described^24^.

### AK2 immunohistochemical examination

The staining patterns were scored as follows: negative, mild (<30% of cells with positive staining), moderate (<60% but more than 30% of cells with positive staining) and strong positive (more than 60% of cells with positive staining) according to the immunostaining intensity. Both moderate and strong positive expressions were categorized into high expression, and negative and mild staining were belonged into low expression.

### siRNA cloning and lentiviral transfection of A549 and H1299 cells

Two shorthairpin RNA (shRNA) against human AK2 gene were 5′-TTCTCCGAACGTGTCACGT-3′, sequence1 (S1) and 5′-CACTCATAGAGTACTACA G-3′, sequence2 (S2), which were subjected to BLAST analysis against the human genome database to eliminate cross-silence phenomena with non-target genes. A scrambled control siRNA (5′-TTCTCCGAACGTGTCACGT-3′) was used as the negative control (sh-NC). The shRNAs were synthesized, annealed and inserted into the lentiviral vectors GV112 (Shanghai Genechem Company Ltd., China) which contains an EGFP (green fluorescent protein) gene as a reporter gene. For lentivirus infection, A549 and H1299 cells were divided into negative control group (sh-NC) and test group (shAK2(S1)/(S2)). The cells were seeded into a six-well plate and incubated at 37 °C with 5% CO_2_ until they reached ~30% confluence before infection. At 72 h post infection, GFP expression was observed under a fluorescence microscope.

### Cell proliferation, cell cycle and cell apoptosis assays

After achieving the logarithmic growth phase, cells transfected with sh-NC or sh-AK2 were digested by trypsin, resuspended in standard medium, and then seeded into 96-well plates at a density of 2,000 cells/well. The number of GFP-positive cells was counted using a Celigo ArrayScan High-Content Screening Reader (Thermo Fisher Scientic, USA) for five consecutive days. To observe the cell cycle distribution and apoptotic ratio, cells transfected with sh-NC or sh-AK2 were incubated in six-well plates for 48 h. Flow cytometer and Hoechest-33342 staining were used to measure the cell cycle and apoptosis of the transfected cells.

### Colony formation assays

Lung adenocarcinoma A549 and H1299 cells (500 cells/per well) in each group were plated in a six-well plate and cultured for ten days. Colonies were then fixed for 5 min with 10% formaldehyde and stained with 0.1% crystal violet for 30 sec. The number of colonies containing 50 cells was counted under a microscope.

### Cell migration and invasion assays

Cell migration and invasion were measured by a 8.0 µm pore size transwell chamber respectively. At 72 h after infection, cells in serum-free media were placed into the upper chamber coated with or without 1 mg/ml matrigel. Media containing 10% FBS were added into the lower chamber. Following 48 h incubation, cells that remained in upper membrane were wiped, while cells that migrated or invaded were fixed in methanol, then stained with 0.1% crystal violet and counted under a microscope (IX71 inverted system microscope, OLYMPUS). The assay was performed as described in^25^.

### Immunofluorescence assay

Cells were processed for immunofluorescence staining as previously described^26^. A tandem monomeric RFP-GFP-tagged LC3 (tfLC3) was used to monitor autophagy activity following manufacturer’s instructions. To evaluate tandem fluorescent LC3 puncta, cells were co-transfected with sh-AK2 or sh-NC after stable transfection with tfLC3, and washed with 1 × PBS at indicated time. Samples were examined and photographed under an IX71 inverted fluorescence microscope system (OLYMPUS).

### Western blotting

Cells were harvested and protein was extracted from cells as previously described^27^. Equal quantities of protein were electrophoresed through a 10% SDS polyacrylamide gel and transferred to a polyvinylidene fluoride (PVDF) membrane (Millipore, Billerica, MA, USA). The membranes were incubated with anti-E-cadherin, Twist, Snail2, Vimentin, LC3I/II, p62 used as the primary antibodies (1:1000 dilution). Following rinsing for 3 times, membranes were incubated with secondary peroxidase-linked goat anti-rabbit IgG (1:5000, CST, USA) for 1.5 h. After washing for 30 min with TBST at room temperature once more, the immunoreactivity was visualized by enhanced chemiluminescence (ECL kit, Pierce Biotechnology), and membranes were exposed to Kodak XAR-5 films (Sigma-Aldrich Chemical). GAPDH was employed as a loading control.

### *In vivo* assays for tumor growth

BALB/c nude mice at 4–6 weeks of age were provided by the Laboratory Animal Research Center of Xinjiang Medical University, and the animal study was reviewed and approved by the Xinjiang Medical University Animal Care and Use Committee. Experimental animals were randomly divided into four groups: (1) sh-NC plus 0.9% normal saline (NS), (2) sh-NC plus hydroxychloroquine (HCQ), an autophagy inhibitor, (3) sh-AK2 plus NS, and (4) sh-AK2 plus HCQ group. A total of 100 μl of suspended cells transfected with sh-AK2 or sh-NC at a concentration of 1 × 105 cells/μl was subcutaneously injected into an unilateral side of the posterior flank of each mouse. Mice were treated with HCQ (60 mg/kg/d) or the same volume of saline solution by peritoneal injection once every 2 days after subcutaneous inoculation for 7 days. Tumor growth was examined every 4 days, and tumor volumes were calculated using the equation V = π/6 × L × W2, where V represents volume, L represents longitudinal diameter, and W represents latitudinal diameter. At 4 weeks post-injection, mice were euthanised, and the subcutaneous growth of each tumor was examined.

### Statistical analysis

Statistical analyses were performed with SPSS version 21.0. χ^2^ test was used to compare the baseline characteristics. Overall survival (OS) was defined as the time to death or last follow-up from the date of diagnosis. Progression-free survival (PFS) was defined as the time to progression or last follow-up from the date of diagnosis for the unresectional cases. According to staining degree, the sample group was categorized into high expression group and low expression group. Survival analysis was performed using the Kaplan-Meier method and log-rank test. Univariate and multivariate analyses using Cox-proportional hazards model were performed to evaluate potential prognostic factors for PFS and OS. For *in vitro* experiment, differences between groups were analyzed using the Student’s t-test. Data were presented as the mean ± SD. A statistically significant difference was defined as p < 0.05.

## Results

### Prognostic significance of AK2 in lung adenocarcinoma

**Figure 1:**
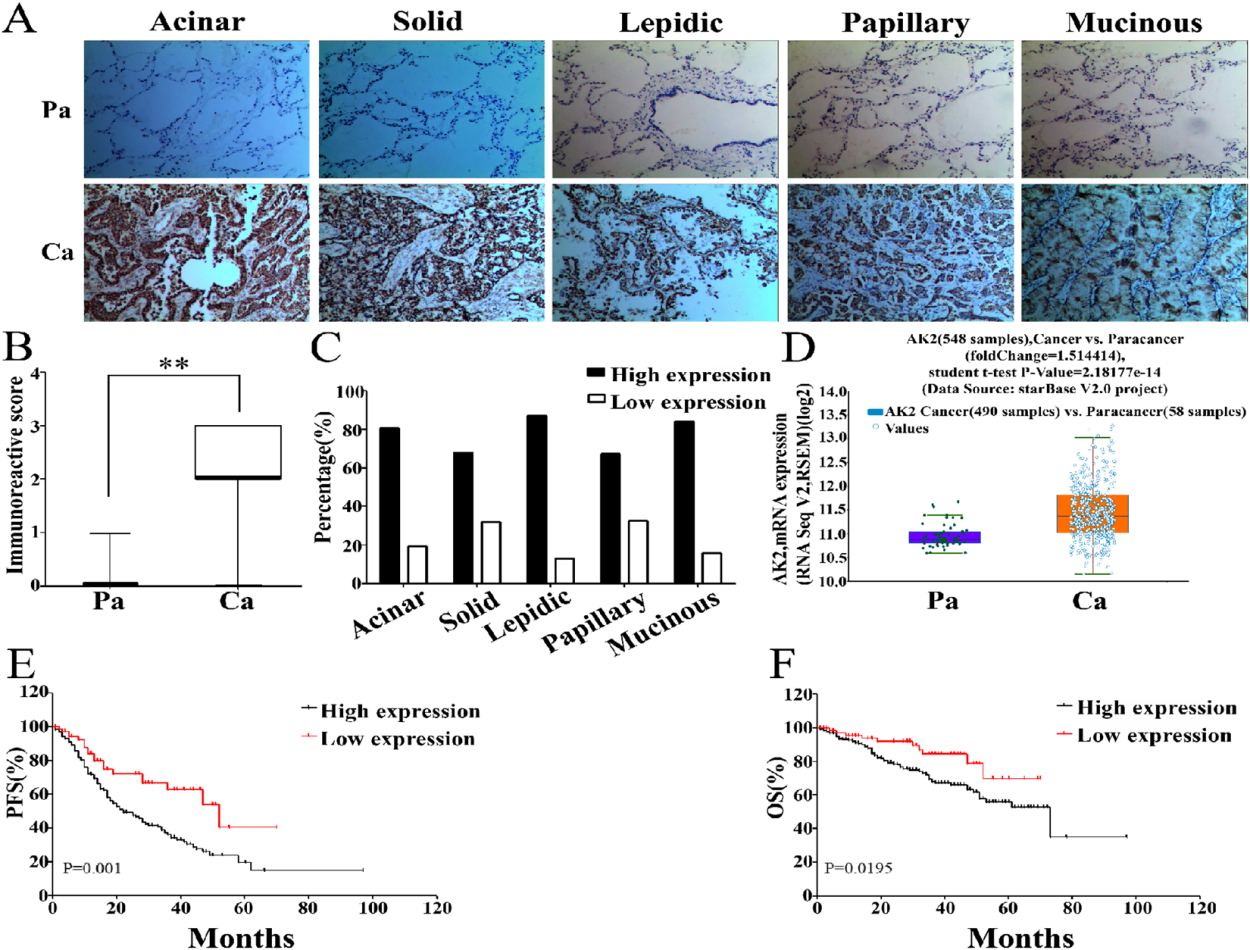
The relative expression of AK2 and prognostic significance in lung adenocarcinoma (LAD). (**A**) Expressionof AK2 in human LAD of different subtypes analyzed with immunohistochemistry (IHC) staining. Representative immunostaining micrographs of AK2 in LAD at IIASLC/ATS/ERS classification were shown. (magnification, ×200). (**B**) Semi-quantitative analysis of the stained sections was performed using light microscopy to calculate the immunoreactive score (see methods). (**C**) Respective percentage of high and low expression of AK2 in five LAD subtypes. (**D**) Relative AK2 expression was analysed by bioinformatics methods using 548 cases of lung cancer (Ca) and paracancerous tissues (Pa) from TCGA database. (**E**) Comparison of PFS between the low expression and high expression groups. (**F**) Comparison of OS between the low expression and high expression groups. Error bars represent the mean ± SD of at least three independent experiments. *p < 0.05, **p < 0.01 vs. control group.

The expression of AK2 was firstly examined by immunohistochemical staining in 345 tumor tissues and 80 adjacent non-tumor counterparts from patients with LAD in our hospital. AK2 protein exhibited a predominantly cytoplasmic staining in lung adenocarcinoma tissues, which was not observed in normal lung tissue, including pneumocytes and other types of stromal cells (Fig. 1A). We divided 345 cases into AK2 high-expression (moderately/strongly positive) group and AK2 low-expression (negative/mildly positive) group. As shown in Fig. 1B,C, AK2 content in the tumor tissues was markedly higher than that in the adjacent non-tumor tissues (P < 0.001). To further confirm AK2 expression in lung adenocarcinoma, we downloaded and analysed the existing clinical data from The Cancer Genome Atlas (TCGA) database including 548 pairs of cancer and paracancerous tissues from LAD. Bioinformatics analysis on the TCGA database demonstrated that the content of AK2 mRNA in LAD tissues was higher than that in adjacent counterparts (Fig. 1D), which were consistent with our conclusions. In terms of clinicopathological correlation analysis, the overexpression of AK2 was significantly correlated with tumor stage (advanced vs early, P < 0.001), lymphatic metastasis (yes vs no, P < 0.001) and histologic subtype (P = 0.007). No significant association was detected between AK2 expression and patient age (≤60 vs >60 years, P = 0.655), gender (male vs female, P = 0.284), tumor size (<3 cm vs ≥3 cm, P = 0.137), smoking (smokers vs non-smokers, P = 0.191), or pleural involvement (yes vs no, P = 0.177) (Table 1). Kaplan-Meier survival analysis showed that patients with high AK2 expression had shorter PFS (P = 0.001) and OS (P = 0.0195) compared to those with low AK2 expression (Fig. 1E,F). Univariate survival analysis indicated that overexpressed AK2 could act as an important prognostic factor for both PFS (P = 0.001) and OS (P = 0.0195), while multivariate survival analysis showed that AK2 expression could predict prognosis independently for PFS (HR: 1.818, 95% CI: 1.126–2.933; P = 0.014) but not an independent risk factor for OS (HR: 1.344, 95% CI: 0.680–2.654; P = 0.395) (Tables 2 and 3). These findings strongly indicated that upregulation of AK2 was frequent in human LAD and might be related to tumor progression.

**Table 2:** Results of the univariate and multivariate analyses of prognostic factors for OS.

Risk factor	Univariate analysis			Multivariate analysis		
	HR*	P value	95% CI	HR*	P value	95% CI
AK2 Expression	2.202	0.019*	1.139–4.258	1.344	0.395	0.680–2.654
Tumor diameter	1.654	0.021*	1.080–2.532	0.948	0.818	0.604–1.489
AJCC stage	4.958	0.001**	3.114–7.894	2.861	0.008**	1.310–6.249
Lymphatic metastasis	4.451	0.001**	2.700–7.335	1.571	0.286	0.685–3.605
pleural metastasis	2.574	0.001**	1.678–3.950	1.746	0.015*	1.113–2.739
Smoking	0.621	0.028*	0.406–0.950	1.455	0.087	0.947–2.234
Gender	1.549	0.040*	1.019–2.356	—	—	—
Age	1.023	0.916	0.673–1.555	—	—	—

HR = Hazard ratio. CI = Confidence interval.

**Table 3:** Results of the univariate and multivariate analyses of prognostic factors for PFS.

Risk factor	Univariate analysis			Multivariate analysis		
	HR*	P value	95% CI	HR*	P value	95% CI
AK2 Expression	2.224	0.001**	1.391–3.555	1.818	0.014*	1.126–2.933
Tumor diameter	1.806	0.001**	1.321–2.469	1.333	0.096	0.950–1.870
AJCC stage	2.526	0.001**	1.847–3.456	1.868	0.016*	1.121–3.113
Lymphatic metastasis	2.162	0.001**	1.570–2.977	1.114	0.685	0.662–1.874
pleural metastasis	1.478	0.014*	1.084–2.015	1.182	0.311	0.856–1.633
Smoking	1.158	0.388	0.830–1.614	—	—	—
Gender	0.937	0.685	0.686–1.281	—	—	—
Age	0.922	0.606	0.677–1.256	—	—	—

HR = Hazard ratio. CI = Confidence interval.

### The knockdown efficacy of AK2

**Figure 2:**
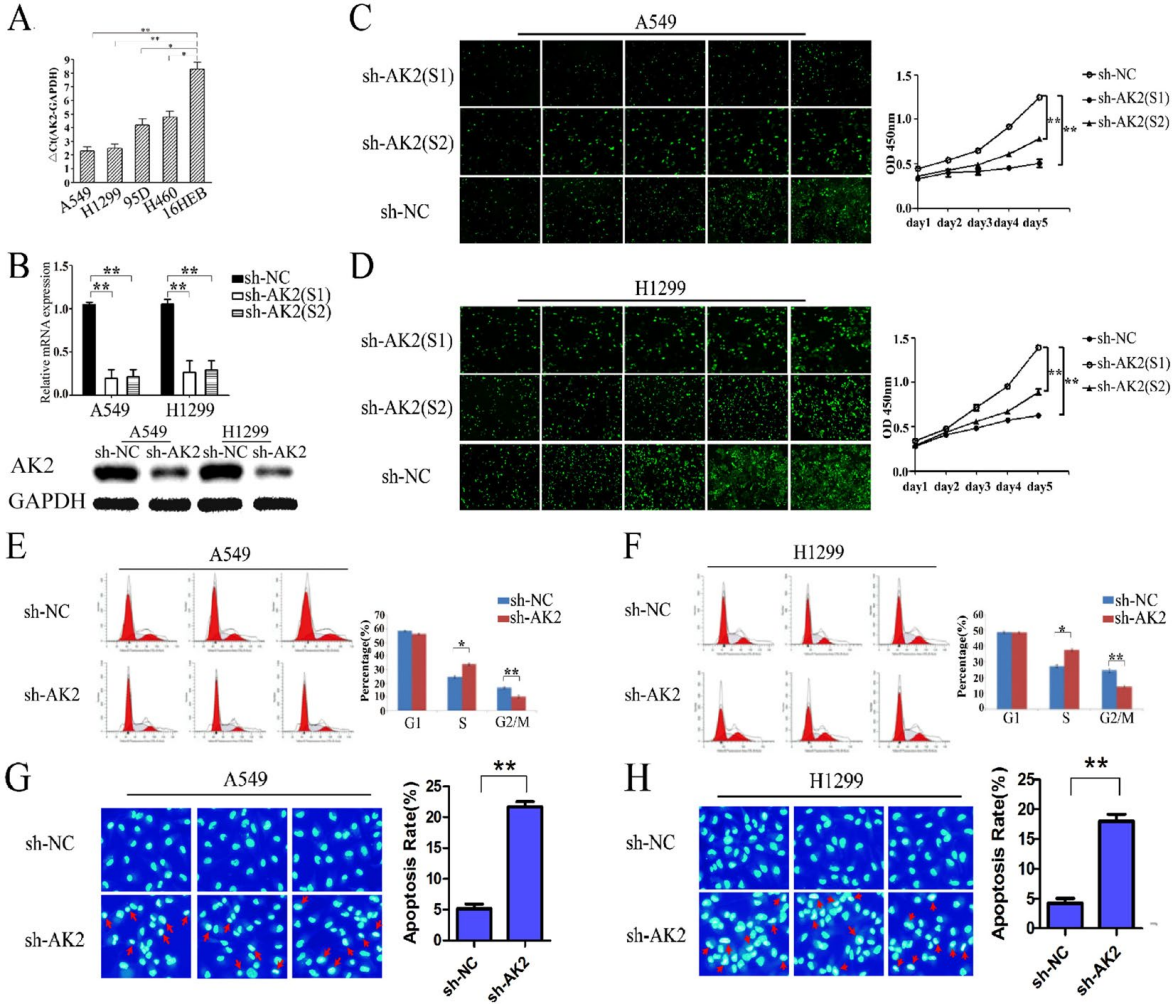
Effects of silencing AK2 on cell proliferation, cycle arrest and apoptosis. (**A**) ΔCt valueof AK2 was examined by qRT-PCR in four LAD cell lines and human normal bronchial epithelial cell line 16HBE. (**B**) Expression of AK2was detected by qRT-PCR in AK2-shRNA (sh-AK2) or scrambled shRNA(sh-NC) transfectants. (**C**,**D**) Impaired cell proliferation in sh-AK2- or sh-NC-transfectedLAD cells was detected by CellomicsArrayScan assay. Growth of A549 and H1299 cells infected by sh-NC (top) or sh-AK2 (bottom) at different time points were shown. (**E**,**F**) Cell cycle analysis was applied to examine the effect of AK2 interference on LAD cellscycle. (**G**,**H**) Flow cytometric analysis was empolyed to detect the effect of AK2 knockdown on LAD cellapoptosis. Error bars represent the mean ± SD of at least three independent experiments. *p < 0.05, **p < 0.01 vs. control group.

AK2 expression was evaluated in four LAD cell lines and a normal bronchial epithelial cell line 16HBE by qRT-PCR. As shown in Fig. 2A, the contents of AK2 were substantially elevated in cancer cells and relatively higher in A549 and H1299 cells among four LAD cell lines. Based on this expression pattern, we therefore chose A549 and H1299 cells for the following loss-of-function studies. To investigate the role of AK2 in LAD, we designed two shRNA sequences to specifically knockdown the expression of AK2 in LAD cell lines A549 and H1299. As shown in Fig. 2B, the levels of AK2 mRNA and protein were significantly reduced following infection of lentivirus, comparing with the sh-NC group (p < 0.001). The original blots have been uploaded as a Supplementary file. The knockdown efficacy of AK2 mRNA in A549 and H1299 cells was 81.5% and 73.4% by sh-AK2(S1) and 78.3% and 70.9% by sh-AK2(S2), respectively (Fig. 2B). Additionally, as shown in Fig. 2C,D, most of the LAD cells were presented EGFP positive signals, suggesting a satisfactory infection efficacy.

### Knockdown of AK2 remarkably inhibited LAD cells growth

To evaluate the function of AK2 on LAD cells growth, we examined the proliferation rate in A549 and H1299 cells using Celigo ArrayScan assay after 5 days of incubation. As shown in Fig. 2C,D, there was a marked inhibition of cell proliferation observed in AK2-silenced A549 and H1299 cells (sh-AK2(S1)/(S2) vs. sh-NC; p < 0.01). Notably, sh-AK2(S1) was more powerful in suppressing cell proliferation than sh-AK2(S2) so sh-AK2(S1) was used in follow-up studies.

### The biological effects of AK2 on LAD cells

**Figure 3:**
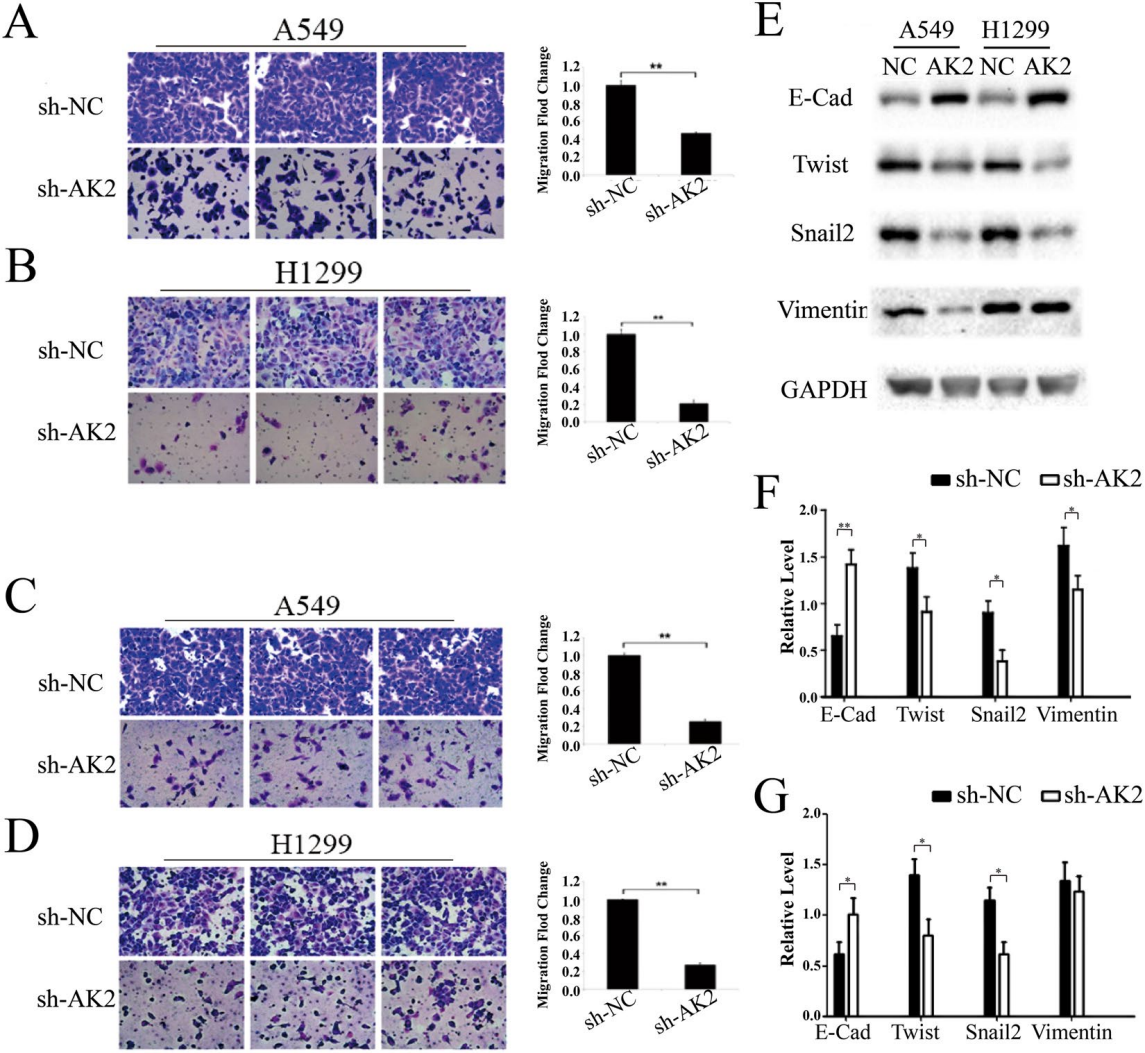
Knockdown of AK2 was associated with cell motility and could reverse EMT to MET of LAD cells. (**A**–**D**) Transwell assays were utilized to exam the function of AK2 on metastasis and invasion ability of LAD cells. Western blotting assays were performed to detect the change of EMT markers in sh-AK2-transfected H1299 and A549 cells. Error bars represent the mean ± SD of at least three independent experiments. *p < 0.05, **p < 0.01 vs. control group. (**E**) Expression of EMT marker was analyzed by western blot in sh-NC- or sh-AK2-transfected A549 and H1299 cells.After 48 h of the transfection, total proteins were isolated, and EMT marker protein was detected by specific antibody. GAPDH was used as an internal control. The grouping of blots were cropped from different gels. (**F**,**G**) Quantification anslyses of EMT marker protein in sh-NC or sh-AK2-transfected A549 and H1299 cells. Error bars represent the mean ± SD of at least three independent experiments. *p < 0.05, **p < 0.01 vs. control group.

To better understand the role of AK2 in LAD tumorigenesis, we examined the cell cycle, apoptotic ratio, motility and EMT in LAD cells. Cell cycle analysis suggested that there was a higher percentage in S phase in sh-AK2-transfected LAD cells when compared to that in control cells (Fig. 2E,F, P < 0.05). Next, the apoptotic ratio was measured by Hoechst 33342 staining. As presented in Fig. 2G,H, silencing AK2 considerably increased the apoptosis ratio of A549 and H1299 cells (P < 0.01) in comparison with that of control group. Moreover, in cell migration assays, downregulation of AK2 markedly reduced the migration of LAD cells, and similar results were obtained in invasion assays (Fig. 3A–D, P < 0.01). To further elucidate whether AK2 regulating cell movement is related to EMT, we examined E-cadherin, a typical epithelial cell marker and the mesenchymal markers, including Vimentin, Snail2 and Twist in stably infected LAD cells. Results showed that the expression of E-cadherin was considerably increased, whereas levels of Snail2 and Vimentin were decreased in both sh-AK2-infected LAD cell lines. However, the expression of Twist was suppressed in A549 cells only but unchanged in H1299 cells infected by sh-AK2 (Fig. 3E–G). The original blots have been uploaded as a Supplementary file. To sum up, expression of AK2 was closely related to apoptosis, cycle arrest, cell movement and EMT in LAD cells.

### AK2 knockdown induced autophagy in LAD cells

**Figure 4:**
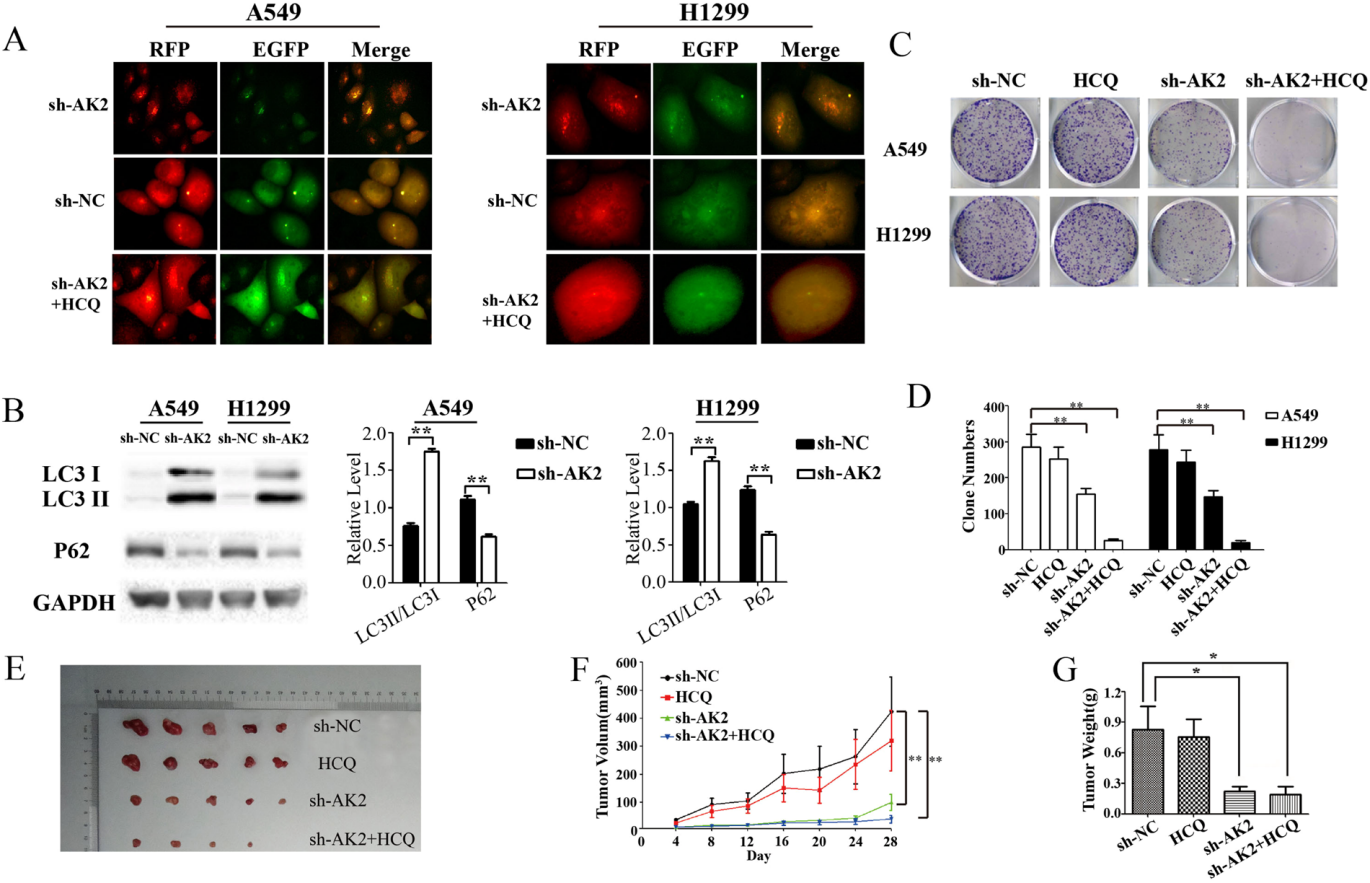
The effects of AK2 knockdown in combination with HCQ on human A549 cells proliferation *in vitro* and in nude mice. (**A**) AK2Knockdown increased punctate LC3 expression in LAD cells. A549 and H1299 cells were respectively co-transfected with an LC3-expressing vector (RFP-GFP-tagged LC3) and sh-NC or sh-AK2. Then cells were fixed and increased green, red, and yellow fluorescent puncta were observed by fluorescent microscopy. (**B**) Changes of autophagosome-associated proteins in LAD cells infected with lentivirus expressing sh-NC or sh-AK2. A549 and H1299 cells were treated for 72 h with sh-NC or sh-AK2 and cell extracts were analyzed by immunoblots with antibodies against LC3 and p62. Blots were also probed for GAPDH.The grouping of blots were cropped from different gels. (**C**) After infected with lentivirus expressing sh-NC or sh-AK2 for 72 h, A549 and H1299 cells were treated with or without 10 µM hydroxychloroquine (HCQ) for 24 h. Colonies were stained with 0.1% crystal violet after an additional 10 days’ culture. (**D**) Quantitative analysis of the number of clones ofsh-NC- or sh-AK2-transfected A549 and H1299 cells combined with or without HCQ. (**E**) Tumors from nude mice in each treatment group. (**F**) Tumor volume in each group. Data are expressed as the mean ± SEM, and were analyzed by 2-way ANOVA. (**G**) Tumor weight in each group. Data were expressed as the mean ± SD, and were analyzed by 2-way ANOVA. Data in B and D are expressed as the mean ± SD, *p < 0.05, **p < 0.01 vs. control group.

To explore whether AK2 deletion could induce autophagy, we further introduced the mRFP-GFP-LC3 reporter to determine the change of autophagic activity caused by AK2 knockdown. When located in autolysosome, this form of LC3 displays only red fluorescence since the GFP signal is sensitive to the acidic condition in the lysosome lumen, whereas the RFP signal is more stable. As seen in Fig. 4A, increased red spots appeared in the merged section of sh-AK2-treated cells and HCQ could effectively reverse AK2 knockdown activated autophagy. In addition, we tested the correlation between AK2 knockdown and autophagy activation by immunoblot. As shown by Fig. 4B, the accumulation of LC3-II increased more in LAD cells infected by sh-AK2 in comparison with that in sh-NC cells. The effects of sh-AK2 was also assessed on the basis of the expression of a second autophagy associated protein, p62, a substrate of the autophagic process that is selectively incorporated into autophagosomes through direct binding to LC3 and is efficiently degraded by autophagy^28^. As expected, p62 expression was greatly decreased in LAD cells after AK2 knockdown. Thus, it can be deduced that there was a high degree of correlation between the activation of autophagy and AK2 deletion in lung cancer cells. The original blots have been uploaded as a Supplementary file.

### Effects of autophagy inhibition on LAD cell proliferation *in vitro* and *in vivo*

Given that autophagy can be activated by AK2 knockdown, we have to ask what role autophagy plays in LAD cells. The most recent reports have shown that the autophagy inhibitor HCQ is tolerable and potentially effective in phase I clinical trials^28,29^. In the present study, HCQ was empolyed to validate the potential function of autophagy *in vitro* and *in vivo*. As expected, the result of clonogenicity tests showed a drastically reduced number of clones in LAD cells co-administrated with silencing AK2 and HCQ when compared to deletion of AK2 or HCQ treatment alone (Fig. 4C,D). To confirm the *in vitro* results, we next investigated the effects of AK2 knockdown in combination with or without HCQ in an animal model. Tumors derived from sh-AK2-infected A549 cells in the presence or absence of HCQ grew more slowly than that derived from sh-NC or treated with HCQ only, and HCQ could notably strengthen anti-proliferative activity of sh-AK2 (Fig. 4E–G). Collectively, these data declared that AK2 interference induced autophagy is cytoprotective. Suppressive effect on LAD cell growth could be strengthened by silencing AK2 in conjunction with autophagy inhibition. Therefore, the combination of AK2 knockdown and HCQ might synergistically improve therapy outcome in lung adenocarcinoma patients.

## Discussion

It is known that AK2 senses AMP, activates metabolic signaling processes and maintains energy homeostasis in the cell. Recent studies have shown that deregulation of AK2 function could be involved in extra-mitochondrial functions, especially in tumorigenesis. In this study, we have reported for the first time that AK2 expression is high in LAD tissues, which is consistent with the statistical analyses on TCGA database. Nevertheless, this finding is in contradiction with a previous report, in which AK was less concentrated in pulmonary tumor tissues than in control lung^22^. However, according to the original literature, specimens used for histological examination just included eight adenocarcinomas, one squamous cell carcinoma, and three large-cell carcinomas, which may have reduced the credibility of the conclusion due to a limited sample volume. There may be other things contributing to the discrepancy, such as ethnic differences, tumor heterogeneity, assay-or platform-specific variables, which are worth considering. Moreover, research by Yong-Keun Jung ‘s team suggests that AK2 expression was drastically downregulated in MCF-7 breast cancer cells and C33A cervical cancer cells and the inoculation of the two tumor cell lines treated with AK2 knockdown into nude mice led to a significant increase of tumorigenesis^21^. However, our study shows that AK2 is an aggressive oncogene in lung adenocarcinoma. Thus, AK2 could play different roles in various neoplastic diseases that is correlated with the tumor tissue origin, extracellular microenvironment and specific mechanisms.

In this study, we observed significant correlation of AK2 expression with clinicopathological characteristics. The immunohistochemical results in the 345 cases of lung adenocarcinoma revealed that expression of AK2 was significantly upregulated in tumors with the advanced stage, larger tumor size, lymph node metastasis, and acinar predominant subtype. Univariate survival analysis revealed that patients in AK2 high-expression group had both shorter PFS (p = 0.001) and OS (p = 0.019) than those in AK2 low-expression group. Both positive AK2 and AJCC stage were independent prognostic factors for poor PFS (p = 0.014 and p = 0.016). Both AJCC stage and pleural invasion were significant indicators of overall survival duration (p = 0.008 and p = 0.015), but there were still no sufficient evidences to support AK2 expression as an independent risk factor for OS (p = 0.395) in univariate analysis. Our findings supported the concept that AK2 overexpression was a marker of increased tumor aggressive behavior.

To further clarify the effects of AK2 in the development of LAD, we determined its expression patterns and biological functions in cultured cells. qRT-PCR analysis showed that the level of AK2 in LAD cells was significantly higher than in normal bronchial epithelial cells. Further, function assays confirmed that knockdown of AK2 could markedly suppress the proliferation, facilitate the apoptosis, inhibit the metastasis ability and reverse the EMT to MET. Particularly, co-administration with HCQ, an effective autophagy inhibitor, could evidently fortify antiproliferative activities of LAD cells stimulated by sh-AK2 *in vitro* and *in vivo*. In tumorigenesis, autophagy can suppress the initiation and development of early tumors, and the loss or inhibition of autophagy promotes aneuploidy and the development of the transformed phenotype^29,30^. However, autophagy can be activated as a pro-survival response that decreases apoptosis caused by toxic subjects under certain conditions^31–33^. To gain insight into the role of autophagy played in cytotoxicity induced by silencing AK2, we evaluated proliferative potentials by virtue of colony forming efficiency and nude mice model. In the case of suppressing AK2 expression combined with HCQ, the colony formation was largely decreased when compared with AK2 knockdown alone. And vivo experiment results also supported this. Thus, it can be inferred that autophagy protected A549 cells from sh-AK2 attack-induced injury. This might be because damaged proteins and organelles making the energy recycling saved cells from death, but inhibition of autophagy blocked the cellular survival mechanism, resulting in more cell death. Another significant observation stemed from our work is that silencing AK2 impairs the invasion and metastasis of LAD cells. The EMT is deemed to be associated with the ability of migration and invasion in cancer cells^34^. In this study, we identified that knockdown of AK2 could induce expression of epithelial marker E-cadherin and suppress levels of the mesenchymal markers. Our findings confirmed that AK2 has a critical role in modulating LAD progression and may be a potential therapeutic target candidate to pulmonary cancer.

## Supplementary information

Supplementary material

## Data Availability

All data generated or analyzed during this study are included in this published article. Raw and processed data are stored in the laboratory of the corresponding authors and are available upon request.
