# Supplementary material for: Prognostic and therapeutic potential of Adenylate kinase 2 in lung adenocarcinoma
Source: Sci Rep. 2019 Nov 28;9:17757. doi: 10.1038/s41598-019-53594-4 (PMC6883075; doi:10.1038/s41598-019-53594-4)
Supplement: Supplementary file 1 — Supplementary material [file 41598_2019_53594_MOESM1_ESM.pdf]

Prognostic and therapeutic potential of Adenylate kinase 2 in lung adenocarcinoma

Huibin Liu[1], Yan Pu[1], Quhai Amina[2], Qiang Wang[2], Mengmeng Zhang[1], Jianzhong Song[1], Jun Guo[1] & Mahmut Mardan[3]

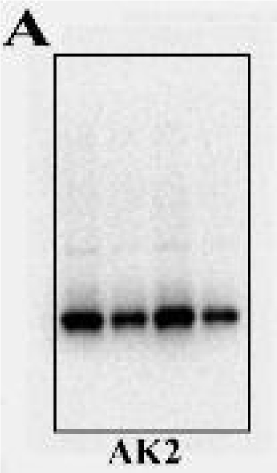

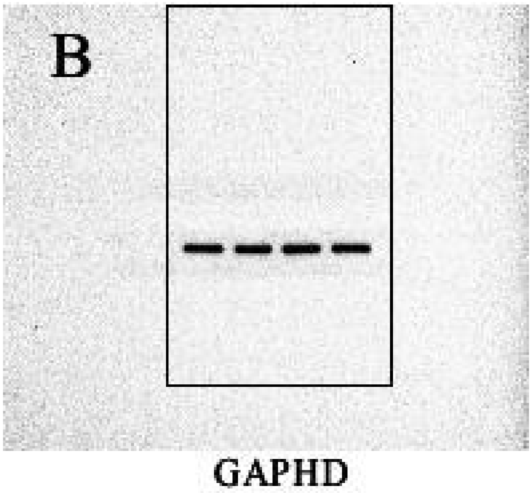

Blots A and B are matched with Figure 2B in our revised manuscript.

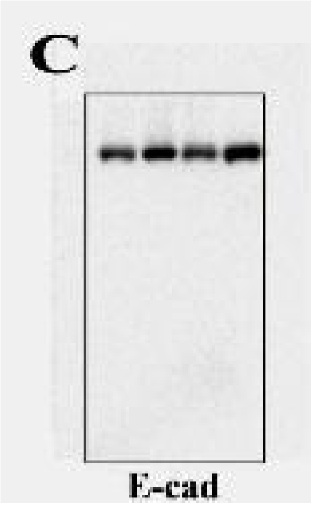

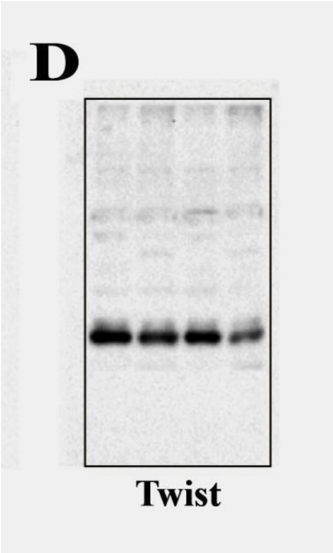

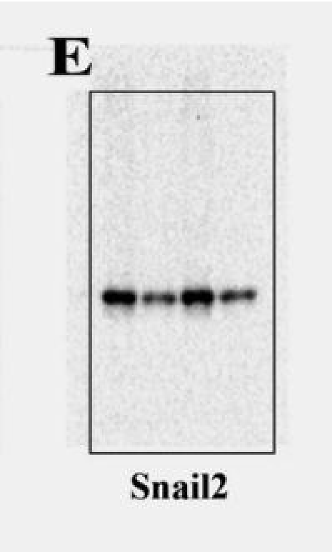

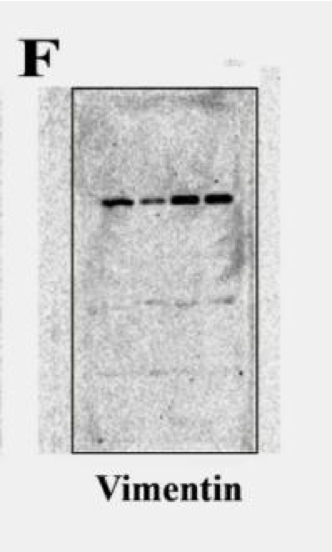

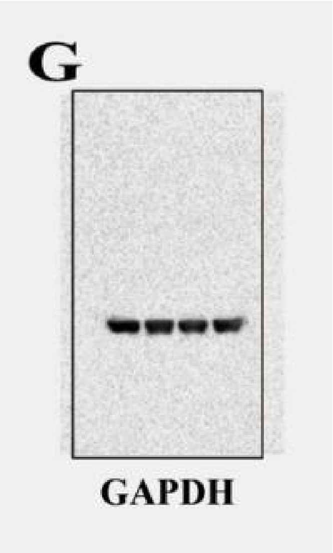

Blots C, D, E, F and G are matched with Figure 3E in our revised manuscript.

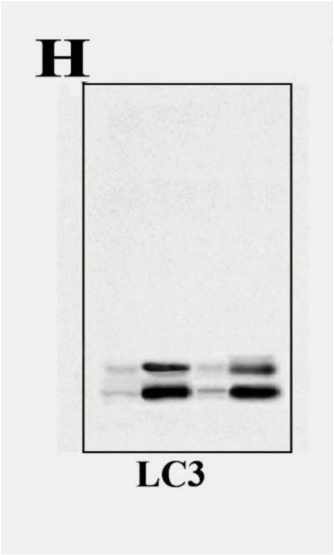

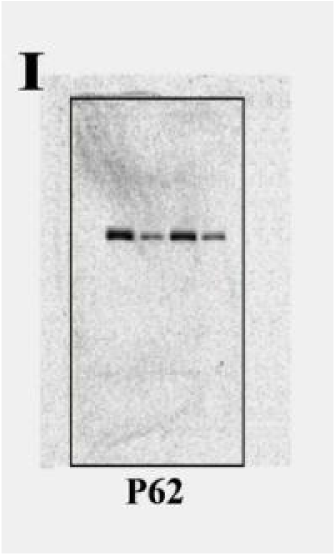

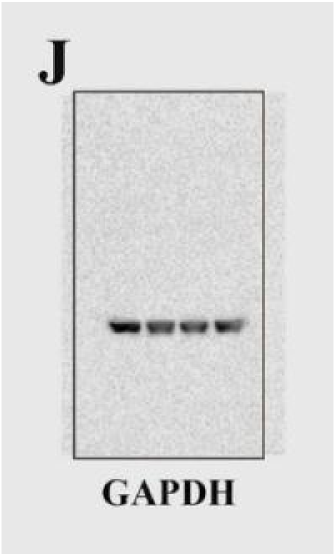

Blots H, I, and J are matched with Figure 4B in our revised manuscript.
